# Supplementary material for: Sex-Dependent Regulation of Placental Oleic Acid and Palmitic Acid Metabolism by Maternal Glycemia and Associations with Birthweight
Source: Int J Mol Sci. 2022 Aug 4;23(15):8685. doi: 10.3390/ijms23158685 (PMC9369035; doi:10.3390/ijms23158685)
Supplement: Supplementary file 1 [file ijms-23-08685-s001.zip › ijms-1797064-supplementary.pdf]

Supplementary 1: Lipid extraction

Frozen placental explants were freeze-dried, weighed, and then lysed using a bead-ruptor homogeniser and phosphate buffered saline (PBS, 1200 µl). Lysate (40 µl) or conditioned media (40 µl) was transferred to an Eppendorf containing 800 µL Butanol/Methanol (1:1) and 10 µl Internal standard mix (Additional file 4). All solvents were LCMS grade and were purchased from Merck except iso-propyl-alcohol which was purchased from Thermo-Fisher Scientific. Samples were briefly vortexed, then sonicated for 30 minutes in an ice bath, then shaken for a further 30 minutes at 4°C. Samples were then centrifuged at 13,000 rpm for 10 minutes, the supernatant transferred to a HPLC tube (*La-Pha-Pack, Germany*) and stored at -80°C. Five quality control samples (BQC) and two blanks were extracted with every placenta. Quality control samples (BQC) were made from placenta lysate pooled from multiple participants, aliquoted and stored at -80 °C.

Supplementary 2: LCMS Methodology

2.1: Sample analysis and LCMS methods

Lipid extracts (5 µl) were injected into an Agilent 6490 triple quadrupole (QQQ) liquid chromatography mass spectrometry instrument alongside a range of standards and analysed as described in Supplementary Methods 3.2. Samples were randomly analyzed with batch quality control samples (BQC) and blanks measured at regular intervals. ^{12}C-PA or ^{12}C-OA lipids were considered quantifiable if BQC percent standard deviation for that lipid was less than 25% and peak area was at least 10x that of the batch blank. ^{13}C-PA or ^{13}C-OA lipids were considered quantifiable if the peak co-eluted with a well quantified ^{12}C endogenous counterpart, and if the peak area was at least 10x that of the batch blank and at least 3x that of placental explant lysate not incubated with ^{13}C-fatty acids. ^{13}C-fatty acid lipid transitions must also give peaks exactly co-eluting with their ^{12}C- fatty acid counterpart, but with an increased mass of 16 (PA lipids) or 18 (OA lipids) Daltons (or multiples if more than one ^{13}C fatty acid is incorporated). Only lipids with no missing data were included in the data set.

Chromatography was performed using a 2.1 x 100 mm 1.8 µm Zorbax Eclipse Plus C18 RRHD (Agilent Technologies) column at 60°C. and the following gradient: Mobile phase A: 50% water, 30% acetonitrile, 20% isopropanol, 10 mmol/L ammonium formate. Mobile phase B: 90% isopropanol, 9% acetonitrile, 1% water, 10 mmol/L ammonium formate. Start (0.4 ml/min): 90% A, 0-2.7minutes: decrease to 55% A, 2.7-2.8 minutes: decrease to 47% A, 2.8-9 minutes: decrease to 35% A, 9-9.1 minutes: decrease to 11% A, 9.1-11 minutes:

decrease to 8% A, 11-11.1 minutes: decrease to 0% A, 11.1 – 11.9 minutes: 0% A, 11.9 – 12 minutes: Increase to 90% A, 12 – 15 minutes: 90% A. The Agilent 6490 triple quadrupole was run with the following settings - Gas temperature: 150 °C. Gas flow: 17 L/min. Nebulizer: 20 psi. Sheath gas temperature: 200 °C. Sheath gas flow: 10 L/min. Positive capillary voltage: 3500 V, Positive nozzle voltage: 1000 V. Positive high pressure RF (iFunnel): 100 V. Positive low pressure RF (iFunnel): 100 V. Fragmentor: 380. Polarity: positive.

2.2 ^{13}C-PA dMRM transition list and internal standard details. Part 1

Lipid	RT	Collision energy	^{12}C-PA		^{13}C-PA		
			Precursor ion	Product ion	Precursor ion	Product ion	Number of ^{13}C-PA
TG_58:9	11.3	21	946.8	673.8	962.8	673.8	1
TG_56:8	11.3	21	920.8	647.8	936.8	647.8	1
TG_56:7	11.4	21	922.8	649.8	938.8	649.8	1
TG_56:6	11.5	21	924.8	651.8	940.8	651.8	1
TG_54:6	11.4	21	896.8	623.8	912.8	623.8	1
TG_54:5	11.4	21	898.9	625.6	928.8	639.8	1
TG_54:4	11.5	21	900.8	627.6	914.9	625.6	1
TG_52:4	11.7	21	872.8	599.6	916.8	627.6	1
TG_52:3	11.45	21	874.8	601.5	888.8	599.6	1
TG_52:2	11.6	21	876.8	603.6	890.8	601.5	1
TG_52:1	11.8	21	878.8	605.5	892.8	603.6	1
TG_51:1	11.9	21	848.8	575.5	894.8	605.5	1
TG_50:2	11.5	21	848.8	575.5	864.8	575.5	2
	11.7	21			882.8	593.5	1
TG_50:1	11.7	21	850.8	577.5	866.8	577.5	2
	11.9	21			884.8	595.5	1
TG_50:0	11.9	21	852.8	579.5	884.8	595.5	2
					868.8	579.5	1
TG_48:1	11.5	21	822.8	549.5	838.8	549.5	1
TG_48:0	11.7	21	824.8	551.5	872.8	583.5	3
	11.7	21			856.8	567.5	2
	11.7	21			840.8	551.5	1
TG_51:0	12.1	21	866.8	579.5	-	-	-

2.3 ^{13}C-PA dMRM transition list and internal standard details. Part 2

Lipid	RT	Collision energy	^{12}C-PA		^{13}C-PA		
			Precursor ion	Product ion	Precursor ion	Product ion	Number of ^{13}C-PA
SM_34:2	5.9	25	701.6	184.1	717.6	184.1	1
SM 30:1	4.5	25	647.5	184.1			
PI_36:4	5.71	17	876.6	599.6	892.6	615.6	1
PI_36:2	6.15	17	880.6	603.6	896.6	619.6	1
PE_36:4	6.9	17	740.5	599.5	756.5	615.5	1
PE_36:3	7.2	17	742.5	601.5	758.5	617.5	1
PE_34:2	6.9	17	716.5	575.5	732.5	591.5	1
PE_34:1	7.65	17	718.5	577.5	734.5	593.5	1
PE_32:0	7.4	17	692.5	551.5	708.5	567.5	1
PE 17:0/17:0	8.6	17	720.6	579.5			
PC_36:4	6.7	21	782.6	184.1	798.6	184.1	1
PC_36:3	6.8	21	784.6	184.1	800.6	184.1	1
PC_34:2	6.8	21	758.6	184.1	774.6	184.1	1
PC_34:1	7.6	21	760.6	184.1	776.6	184.1	1
PC_32:0	7.3	21	734.6	184.1	750.6	184.1	1
PC 13:0/13:0	4.751	21	650.5	184.1			
LPC_16:0	2.75	21	496.3	184.1	512.3	184.1	1
LPC 13:0	1.75	21	454.3	184.1			
Hex1Cer_d18:1.16:0	6.55	33	700.6	264.3	716.6	264.3	1
DG_38:4	10.064	21	662.6	389.3	678.6	389.3	1
DG_36:3	9.9	21	636.6	363.3	652.6	363.3	1
DG_34:2	9.6	21	610.5	337.2	626.5	337.2	1
DG_32:0	10	21	586.5	313.2	618.5	329.2	2
					602.5	313.2	1
Cer_d18:2_16:0	6.98	23	536.5	262.3	552.5	262.3	1
Cer_d18:1_16:0	6.733	29	538.5	264.3	526.6	236.3	1
dhCer 8:0	4.722	31	428.4	284.3			
Acylcarn_18:0	3.426	30	428.4	85.1	444.4	85.1	1
Acylcarn_16:0	2.72	30	400.4	85.1	416.4	85.1	1
Acylcarn_14:0	1.99	30	372.3	85.1	386.3	85.1	1
Acylcarn_12:0	1.35	30	344.3	85.1	356.3	85.1	1
AcylCarn 16:0 d3	2.7	30	403.3	85.1			

2.4 ^{13}C-OA dMRM transition list and internal standard details

Lipid	RT	Collision energy	^{12}C-OA		^{13}C-OA		
			Precursor ion	Product ion	Precursor ion	Product ion	Number of ^{13}C-PA
TG_58:9	11.2	21	946.8	647.5	964.8	647.5	1
TG_58:10	11.6	21	944.8	645.5	962.8	645.5	1
TG_56:8	11.7	21	920.8	621.5	938.8	621.5	1
TG_56:6	11.4	21	924.8	625.5	942.8	625.5	1
TG_54:7	11.7	21	894.8	595.5	912.8	595.5	1
TG_54:5	11.5	21	898.9	599.6	916.9	599.6	1
TG_54:4	11.5	21	900.9	601.6	936.9	619.6	2
					918.9	601.6	1
TG_54:3	11.7	21	902.9	603.6	956.9	639.6	3
					938.9	621.6	2
					920.9	603.6	1
TG_54:2	11.9	21	904.9	605.6	922.9	605.6	1
TG_52:4	11.35	21	872.8	573.5	890.8	573.5	1
TG_52:3	11.5	21	874.8	575.5	892.8	575.5	1
TG_52:2	11.7	21	876.8	577.5	894.8	577.5	1
TG_52:1	11.88	21	878.8	579.5	896.8	579.5	1
TG_51:0			866.8	579.5			
TG_50:3	11.3	21	846.8	547.5	864.8	547.5	1
TG_50:2	11.5	21	848.8	549.5	866.8	549.5	1
TG_50:1	11.7	21	850.8	551.5	868.8	551.5	1
TG_50:0	11.7	21	852.8	553.5	870.8	553.5	1
TG_48:3	11.1	21	818.8	519.5	836.8	519.5	1
TG_48:1	11.55	21	822.8	523.5	840.8	523.5	1
PS_36:1	7.5	25	790.6	605.6	808.6	623.6	1
PI_34:1	6.3	17	854.6	577.6	872.6	595.6	1
PE_40:7	6.8	17	790.5	649.5	808.5	667.5	1
PE_38:5	7.6	17	766.5	625.5	784.5	643.5	1
PE_36:2	7.938	17	744.6	603.5	780.6	639.6	2
PE_36:2	7.938	17			762.6	621.6	1
PE_36:1	9.1	17	746.6	605.6	764.6	623.6	1
PE_34:1	7.6	17	718.5	577.5	736.5	595.5	1
PE-P 16:0_18:1	8.2	17	702.5	339.3	720.5	357.3	1
PE 17:0/17:0	7.6	17	720.6	579.5			
PC_38:4	7.87	21	810.6	184.1	828.6	184.1	1
PC_34:1	7.6	21	760.6	184.1	778.6	184.1	1
PC 13:0/13:0			650.5	184.1			1
LPE_18:1	3	17	480.3	339.3	498.3	357.3	1
LPE 14:0			426.3	285.2			
LPC_18:1	3	21	522.4	184.1	540.4	184.1	1
LPC 13:0			454.3	184.1			
DG_38:4	10.3	21	662.6	363.3	680.6	363.3	1
Acylcarn_18:1	2.959	30	426.4	85.1	444.4	85.1	1
Acylcarn_16:1	2.276	30	398.3	85.1	414.3	85.1	1
Acylcarn_14:1	1.6	30	370.3	85.1	384.3	85.1	1
AcylCarn 16:0 d3			403.3	85.1			

Supplementary 3: Data analysis and statistics

Metabolite peak areas were integrated using Mass Hunter QQQ Quantitative Analysis Version 8. Metabolite peaks were quantified using the following internal standards: TG - TG_51:0 (Sigma Aldrich, 85.1 pmol per sample). PE, PS and PI - PE 17:0/17:0 (Avanti Polar lipids, 100.7 pmol per sample). PC - PC 13:0 13:0 (Avanti Polar lipids, 72.1 pmol per sample), LPC - LPC 13:0 (Avanti Polar lipids, 107 pmol per sample). LPE - LPE 14:0 (Avanti Polar lipids, 114.1 pmol per sample). Ceramides and dihydroceramides - dhCer 8.0 (Avanti Polar lipids, 56.3 pmol per sample). Acylcarnitine - Acylcarnitine 16:0 d3 (Avanti Polar lipids, 914 pmol per sample). Sphingomyelin - SM 30:1 (Avanti Polar lipids, 96.39 pmol per sample). Data was analyzed in R version 4.1.1 (Kick Things) using the tidyverse, tidymodels dplyr, purrr, broom, readr, moderndive, rstatix, dlookr packages. Graphs were made using the ggplot2, ggpubr, ggrepel, ggforce, ggthemes and viridis packages. Only lipids with no missing values below the limits of quantification were included in the dataset. Peak areas were normalized against their corresponding lipid class internal standard to give concentration expressed as pmol/ml. Dried placental mass was used to calculate the lipid amount in explants expressed as pmol/ dry mg placenta using the following calculations:

Amount of lipid in sample = Peak area lipid/ Peak area internal standard*amount of internal standard in sample

Proportion of lysate used to prepare sample = 40 µl / total lysate (1200 µl) = 30

Amount of lipid in lysate = Amount of lipid in sample*30

Amount of lipid per mg dry placenta = Amount of lipid in lysate/ mass dry placenta used to prepare lysate
